# Exposure to Endocrine Disrupting Chemicals in the Dutch general population is associated with adiposity-related traits

**DOI:** 10.1038/s41598-020-66284-3

**Published:** 2020-06-09

**Authors:** Thomas P. van der Meer, Martijn van Faassen, André P. van Beek, Harold Snieder, Ido P. Kema, Bruce H. R. Wolffenbuttel, Jana V. van Vliet-Ostaptchouk

**Affiliations:** 1 Department of Endocrinology, University of Groningen, University Medical Center Groningen, Groningen, the Netherlands; 2 Department of Laboratory Medicine, University of Groningen, University Medical Center Groningen, Groningen, the Netherlands; 3 Department of Epidemiology, University of Groningen, University Medical Center Groningen, Groningen, The Netherlands; 4 Genomics Coordination Center, Department of Genetics, University of Groningen, University Medical Center Groningen, Groningen, The Netherlands

**Keywords:** Obesity, Environmental impact, Metabolic syndrome

## Abstract

Endocrine Disrupting Chemicals (EDCs) have been linked to a variety of cardiometabolic diseases. Yet, few studies have investigated the exposure to EDCs and cardiometabolic health taking lifestyle into account. We aimed to assess exposure to five parabens, three bisphenols and thirteen metabolites of in total eight phthalates in a general Dutch population and to investigate their association with cardiometabolic traits. In 662 adult subjects from the population-based Lifelines cohort, 21 EDC analytes were measured in 24-hour urine collected in 2012, using LC-MS/MS. Association analyses between cardiometabolic traits and EDC concentrations were performed using multivariate linear models adjusting for age, sex, education, smoking, diabetes, physical activity and caloric intake. Quartile analyses were performed to assess linearity. Bisphenol A, four parabens and eight phthalate metabolites were detected in 84-100% of the samples. Adjusted associations for MiBP and MBzP and adiposity-related traits were robust for multiple testing (Beta’s, BMI: 1.12, 2.52; waist circumference: 0.64, 1.56, respectively; FDR < 0.009). Associations for triglyceride, HDL-cholesterol, glucose and blood pressure were not. Linearity was confirmed for significant associations. Exposure to EDCs in the Dutch population is ubiquitous. We found direct associations between phthalates and adiposity-related traits. Prospective studies are needed to confirm these findings.

## Introduction

During the past decades, cardiovascular diseases (CVD) and type 2 diabetes (T2D) have risen to epidemic proportions^1,2^. These diseases are responsible for severe complications including myocardial infarction, stroke, blindness, lower limb amputation and renal failure, and are strongly correlated with a collection of asymptomatic cardiometabolic abnormalities such as (central) obesity, impaired glucose tolerance, elevated triglycerides, a low HDL-cholesterol, and hypertension^3,4^. A combination of at least three of the above stated cardiovascular abnormalities defines the metabolic syndrome (MetS)^1^. Risk factors include a combination of genetic predisposition and lifestyle (e.g. lack of physical activity, imbalanced diet).

Meanwhile, a wide variety of synthetic chemicals have been introduced in our environment, some of which have been shown to cause metabolic disruptions in animal and human studies^5^. Endocrine Disrupting Chemicals (EDCs) such as parabens, phenols and phthalates have been shown to be associated with the MetS^6,7^, obesity^8,9^ and T2D^10,11^. These EDCs have in common that they are widely used as preservatives and plasticizers and can therefore be found in a wide variety of consumer products. Exposure occurs through ingestion, inhalation, and dermal contact^12,13^. Although parabens, phenols and phthalates have in common that they are metabolized and excreted fairly quickly (i.e. half-lives <24 h) and therefore are considered non-persistent^14–16^, exposure is ubiquitous throughout life and reported to be global^17–20^. However, to date little is known about exposure to these EDCs in the general Dutch population.

Common parabens, such as methyl paraben (MeP), ethyl paraben (EtP), propyl paraben (PrP), n-butyl paraben (n-BuP) and benzyl paraben (BzP) are widely used as antimicrobial preservatives in food, cosmetics, personal care products (e.g. soaps, makeup, lotions and sanitary products) and pharmaceuticals. These compounds are known to possess weak estrogenic properties. Adverse effects of parabens on lipid metabolism have been described *in vitro* and *in vivo*^21,22^. Bisphenol A (BPA) is a chemical that is produced in one of the highest volumes worldwide. It can be found in a wide range of products such as plastic bottles and food packaging, personal care products and paper receipts. BPA has weak estrogenic and anti-androgenic effects^23,24^. and has been associated with adverse effects on T2D^25,26^, CVD^25^, obesity^27^, and the MetS^26^. Due to the restriction of BPA in children’s toys by the European Union and growing consumer concern, BPA is increasingly being replaced by analogues such as Bisphenol F (BPF) and Bisphenol S (BPS) creating “BPA-free” products^28^. Yet, these analogues appear to be at least as deleterious for the endocrine system as BPA^29^. Phthalates are known for their anti-androgenic effects and can be divided in Low-Molecular-Weight (LMW) and High-Molecular-Weight (HMW) phthalates. LMW-phthalates are most commonly used in food products, cosmetics, personal care products and medications, whereas HMW-phthalates are used as soft plastics in food packaging, medical tubing and toys^30–32^. Associations of phthalates have been found with high body mass index (BMI) and waist circumference^9^, high blood pressure^7,33^, (pre-)diabetes^10,11^, and the MetS^6^. Yet, associations of parabens, bisphenols and phthalates with cardiometabolic outcomes are not consistently observed in epidemiological studies. Next to positive findings in the studies mentioned above, other studies have not been able to confirm these associations^34–37^.

In this study, we assessed the exposure to the most common non-persistent EDCs including five parabens, three bisphenols and thirteen metabolites of in total eight different phthalate diesters in a general Dutch population and investigated potential associations between these EDCs and cardiometabolic traits.

## Methods

### Study population

This study consisted of 662 native Dutch adult subjects randomly selected as a subsample from the Lifelines Cohort Study with available data on physical examination, biochemical measurements, extensive questionnaires, and 24-hour (24 h) urine samples. Lifelines is a population-based cohort study, of which the cohort profile has been described elsewhere^38^, and is representative for the north of the Netherlands^39^. The Lifelines Cohort Study is conducted in accordance with the Declaration of Helsinki and the research code of the University Medical Center Groningen (UMCG). Before study entrance, participants signed an informed consent. The study was approved by the UMCG medical ethics review committee.

### Clinical measurements

In Lifelines, measurements were performed by a trained research nurse using a standardized protocol. Height, weight and waist circumference were measured to the nearest 0.5 cm, 0.1 kg, and 0.5 cm, respectively. Waist circumference was measured in standing position, with a tape measure at the level midway between the lower rib margin and the iliac crest. Blood pressure was measured automatically (Dinamap PRO 100V2) in supine position every minute for 10 minutes, after which the average of the last three measurements was taken for both systolic and diastolic blood pressure. BMI was calculated as (weight (kg)/height (m)^2^). Per individual, blood was collected in fasting state and glucose, triglycerides and HDL- cholesterol measurements were performed on the same day. Glucose (mmol/L) was measured using a hexokinase method. HDL-cholesterol (mmol/L) and triglycerides (mmol/L) were measured using an enzymatic colorimetric method and a colorimetric UV method, respectively, using a Roche Modular P chemistry analyser (Roche, Basel, Switzerland). We aimed to measure EDC exposure which is representative for daily exposure. Therefore, participants collected all their urine over a period of 24 h during normal living conditions without any specific dietary instructions. Containers were specifically supplied for this purpose and were accompanied by oral and written instructions. Aliquots of urine were stored at −80 °C for later laboratory assessment^38^.

### Assessment of potential confounding factors

Extensive information was gathered using questionnaires on demographic characteristics and lifestyle as described somewhere else^38^. Smoking status and Type 2 Diabetes diagnosis were assessed using a questionnaire and categorized as yes or no (reference group). Education level was measured according to the International Standard Classification of Education with a single-item question on the highest educational level achieved^40^, and classified as low (no education, primary education, lower or preparatory vocational education, or lower general secondary education), medium (intermediate vocational education or apprenticeship, or higher general secondary education or pre-university secondary education) or high (reference group) (higher vocational education, or university). Physical activity was assessed using the short questionnaire to assess health-enhancing physical activity (SQUASH), which is extensively described elsewhere^41^. In short, it includes questions on time and effort of activities including commuting, leisure-time, household, work and school. A total activity score is calculated by multiplying the total minutes of activity per week by its intensity score. Caloric intake was calculated based on the Lifelines Diet Score, a 110-item food frequency questionnaire, and expressed as total amount of kilocalories per day over the previous month and is therefore a general average^42^.

### Paraben, bisphenol and phthalate measurements and 24 h calculations

The parabens MeP, EtP, PrP, n-BuP and BzP, the bisphenols BPA, BPF and BPS and metabolites of dimethyl phthalate (DMP), diethyl phthalate (DEP), di-iso-butyl phthalate (DiBP), di-n-butyl phthalate (DnBP), di-(2-ethyl-hexyl) phthalate (DEHP), butylbenzyl phthalate (BBzP), di-iso-nonyl phthalate (DiNP), and di-iso-decyl phthalate (DiDP) were analysed in 24 h urine samples using two offline isotope dilution liquid chromatography tandem mass spectrometry (LC-MS/MS) technology, for which the full analytical methods have been described elsewhere^43^. In short, the aliquot was homogenized before subsamples were taken. For both methods a Phenomenex Kinetex Phenyl-Hexyl 2.1 × 100 mm, 1.7 μm was used at 40 °C in combination with a Waters XEVO TQ-S triple quadrupole system using negative electrospray ionization. Transitions and qualitative and quantitative ions have been published earlier^43^, and are reproduced here in Supplementary table 1a and 1b for completeness. Stable isotope labelled internal standards were used (when available 13 C labelled, otherwise deuterium labelled). For phenols, the mobile phase consisted of 0.2 mM ammonium fluoride in 10% methanol in water (buffer A) and methanol (buffer B). For phthalates, 0.1% formic acid in 10% acetonitrile was used as mobile phase A and 0.1% formic acid in acetonitrile as mobile phase B. The flow rate for both methods was 0.4 mL/min. Runtimes were 7 minutes and 8 minutes per sample for the phenol and phthalate method, respectively.

A limit of detection (LOD) was calculated as “3.3*S0/b”, where S0 is the standard deviation of the response and b the slope of the calibration curve. The limit of quantification (LOQ) was set where the imprecision was ≤20% and the signal to noise ratio was >10 on six days. Urinary excretions of EDCs per 24 h (ng/24 h) were calculated by multiplying the measured concentrations of EDCs (ng/mL) by the total urinary volume (mL) of collected urine in 24 h. This way, we corrected for liquid intake and urine dilution. To assess total exposure of parabens, bisphenols and phthalates, aggregated concentrations were calculated. First measured compounds were converted to molar concentrations by dividing the measured total excreted EDCs per 24 h with the respective molecular weight and expressed in nmol, using the formula: “molar concentration = raw *(1/molecular weight) *10^3”. Subsequently, phthalate molar concentrations were calculated by the sum of their respective metabolites (DEHP = MEHP + MnHP + MEHHP + MEOHP + MECPP; DiNP = MiNP + MHiNP). Phthalates with a single metabolite are expressed as its metabolite (DMP as MMP, DEP as MEP, DiBP as MiBP, DnBP as MnBP, BBzP as MBzP, and DiDP as MiDP). In addition, the respective phthalates were added to calculate the concentration of LMW-phthalates (DMP, DEP, DiBP and DnBP) and HMW-phthalates (DEHP, BBzP, DiNP, and DiDP). Total bisphenol exposure was calculated by summing its molar concentrations (BPA, BPF and BPS), as was done for total paraben exposure (MeP, EtP, PrP, n-BuP and BzP).

### Analytical approach

Exposure to EDCs was assessed by the number of individuals in which EDC concentrations were above the LOD and are shown as ratio of the full population (i.e. number of individuals with EDC concentration >LOD/total population * 100%). Concentrations and distribution of raw and total excreted EDCs per 24 h were shown as 25^th^, 50^th^ (median), 75^th^ quartile and maximum.

Next, we aimed to test whether exposure to EDCs was associated with cardiometabolic traits. As the LOQ indicates a threshold at which an analyte can be precisely measured, only EDCs which were detected above LOQ in at least 50% of the samples (i.e. EDC > LOQ in n > 331) were included in these analyses. For concentrations between LOD and LOQ, the value generated by the LC-MS/MS was used. Concentrations below LOD were imputed with the LOD divided by the square root of two (LOD/√2). Assumptions of linear regression were tested before analysis. Due to a right-skewed distribution, all EDCs and triglycerides were log_10_-transformed before analyses. Fasting glucose can be affected by diabetes. Therefore, individuals which reported being diabetic (n = 8), or with a fasting glucose which was ≥7.0 mmol/L (n = 41) were excluded from association analyses for EDCs and glucose. Associations were tested using two multivariate linear regression models. As EDCs have shown to differ between age and sex^10,44^, these factors have been adjusted for in the base model. Further, we assessed the robustness of associations by additionally correcting for factors known to be associated with EDC concentrations or cardiometabolic traits in the full model. EDC concentrations have been shown to differ between level of education, diabetes and smoking status^10,45–47^. Further, the effect of lifestyle on cardiometabolic traits was taken into account in the full model by adjusting for caloric intake and physical activity.

Some associations between EDCs and cardiometabolic traits have been shown to follow a nonmonotonic dose-response curve^48,49^. In order to accommodate potential non-linear effects and test the robustness of our analysis, we categorized EDC exposure into quartiles and repeated the full multivariate linear model. In these quartile analyses, the quartile including individuals with the lowest exposure (quartile 1) was taken as reference group.

As this study includes many different EDCs, multiple testing was taken into account using Benjamini and Hochberg False Discovery Rate (FDR < 0.05) for all individual tests within the respective cardiometabolic trait. All analyses were performed in R software version 3.5.3^50^.

## Results

### Detection of endocrine disrupting chemicals in a general dutch population

The study population consisted of slightly more females (58%) with a mean age of 46 years. Regarding the criteria for the MetS, mean values of cardiometabolic traits were well within the healthy range (Table 1). Concentrations of detected EDCs in Lifelines are shown in Table 2. The parabens MeP, EtP, and PrP, were detected above LOD in ≥93% of the subjects, whereas n-BuP and BzP were detected in 86% and 5%. of the cases, respectively. Of the bisphenols, BPA was detected in 95% of the samples, followed by BPF (52%) and BPS (9%). Of the phthalate metabolites, MEP, MiBP, MnBP, MEHHP, MEOHP, MECPP and MBzP were detected in all samples. MEHP, MMP, MnHP, MiDP, and MiNP were detected in 84%, 53%, 21%, 10% and 1% of the samples, respectively. MHiNP was detected in none of the samples and is therefore not displayed. Only EDCs detected above LOQ in >50% of the population were included in further analyses (phenols: MeP, EtP, PrP, and BPA; phthalates: MEP, MiBP, MnBP, MEHP, MEHHP, MEOHP, MECPP, and MBzP).

**Table 1:** Characteristics of the study population drawn from the Lifelines cohort (n = 662).

Characteristics	
Sex = Male (%)	280 (42)
Age (years)	45.8 (13)
BMI (kg/m^2^)	24.8^23,28^ *
Waist circumference (cm)	89.8 (12)
Fasting Glucose (mmol/L)	5.0 (0.6)
Triglycerides (mmol/L)	0.92 [0.7, 1.3]*
HDL Cholesterol (mmol/L)^♂^	1.36 (0.3)
HDL Cholesterol (mmol/L)^♀^	1.68 (0.4)
Diastolic BP (mmHg)	71 (9.0)
Systolic BP (mmHg)	120 (13)
Smoking (yes, %)**	114 (17)
Type 2 diabetes (yes, %)	8 (1)
Education level***	
Low	119
Medium	237
High	292

Values are expressed as mean (standard deviation) or median [inter-quartile range]. *non-normal distribution, given as median [interquartile range]; ** NA:9; *** NA: 14; ^♂^male, ^♀^female. Glucose and lipids are in expressed in mmol/L. Abbreviations: SD, standard deviation; BMI, Body Mass Index; LDL, Low-Density Lipoprotein; HDL, High-Density Lipoprotein; BP, Blood Pressure; NA, not available.

**Table 2:** Urinary excretion of individual Endocrine Disrupting Chemicals, and grouped Endocrine Disrupting Chemicals.

	Abbreviation	N > LOD (%)	N > LOQ (%)	Raw values (ng/mL)		Volume-adjusted values (µg/24 h)	
				Median [Q25, Q75]	max	Median [Q25, Q75]	max
**Parabens (nmol)**						437 [100; 1259)	75420
Methyl paraben	MeP	662 (100)	654 (99)	26.85 [5.8; 76]	4079	48.5 [10; 129]	8134
Ethyl paraben	EtP	649 (98)	507 (77)	1.68 [0.53; 7.3]	488	2.95 [1.0; 12.7]	620
Propyl paraben	PrP	617 (93)	427 (65)	2.70 [0.5; 20]	1963	4.61 [0.8; 37]	3914
n-Butyl paraben	n-BuP	571 (86)	135 (20)	0.16 [0.1; 0.7]	64.5	0.29 [0.1; 1.2]	188
Benzyl paraben	BzP	36 (5)	0 (0)	<LOD [ < LOD; < LOD]	0.71	<LOD [ < LOD; < LOD]	3.37
**Bisphenols (nmol)**						19.8 [9.96; 36.2]	574
Bisphenol A	BPA	628 (95)	410 (62)	1.9 [0.96; 3.63]	54.4	3.31 [1.6; 6.5]	130
Bisphenol F	BPF	342 (52)	85 (13)	0.24 [<LOD; 0.68]	56.4	0.28 [<LOD; 1.2]	78.2
Bisphenol S	BPS	59 (9)	10 (2)	<LOD [ < LOD; < LOD]	4.06	<LOD [ < LOD; < LOD]	9.64
**Phthalates**							
**Low Molecular Weight-phthalates (nmol)**					880 [504; 1727]	47154	
Mono-methyl phthalate	MMP	349 (53)	76 (11)	0.48 [<LOD; 1.2]	37.6	0.66 [<LOD; 1.9]	38.1
Mono-ethyl phthalate	MEP	661 (100)	661 (100)	47.1 [20; 132]	6635	83.5 [34; 247]	8950
Mono-iso-butyl phthalate	MiBP	662 (100)	662 (100)	19.7 [12; 34]	389	33.0 [24; 51]	493
Mono-n-butyl phthalate	MnBP	662 (100)	662 (100)	17.5 [11; 29]	367	31.0 [20; 45]	760
**High Molecular Weight-phthalates (nmol)**					220 [153; 305]	4798	
**Di-(2-ethyl-hexyl) phthalate (nmol)**	**DEHP**					165 [118; 229]	4737
Mono-(2-ethylhexyl) phthalate	MEHP	556 (84)	343 (52)	2.07 [1.0; 3.7]	50.5	3.96 [1.7; 6.3]	104
Mono-n-hexyl phthalate	MnHP	140 (21)	23 (3)	<LOD [ < LOD; < LOD]	18.2	<LOD [ < LOD; < LOD]	30.9
Mono-(2-ethyl-5-hydroxyhexyl) phthalate	MEHHP	662 (100)	662 (100)	9.12 [6.2; 14]	183	16.0 [11; 23]	377
Mono-(2-ethyl-5-oxohexyl) phthalate	MEOHP	662 (100)	660 (100)	6.15 [4.1; 9.6]	146	11.0 [7.6; 16]	301
Mono-(2-ethyl-5-carboxypentyl) phthalate	MECPP	662 (100)	662 (100)	10.3 [6.6; 16]	307	17.59 [12; 25]	633
Mono-benzyl phthalate	MBzP	662 (100)	639 (97)	5.84 [3.3; 11]	617	9.95 [5.9; 18]	360
Mono-iso-nonyl phthalate	MiNP	5 (1)	4 (1)	<LOD [ < LOD; < LOD]	5.31	<LOD [ < LOD; < LOD]	13.9
Mono-hydroxy-iso-nonyl phthalate	MiDP	63 (10)	2 (0)	<LOD [ < LOD; < LOD]	2.90	<LOD [ < LOD; < LOD]	7.58

Abbreviations: h, hour; LOD, limit of detection; LOQ, limit of quantification; min, minimum (lowest detected concentration); Q25, 25th quartile; Q75, 75th quartile; max, maximum (highest detected concentration). Total concentrations of parabens, bisphenols, low molecular weight- and high molecular weight-phthalates (including DEHP) were calculated by summing the molar concentrations of its respective chemicals. Mono-hydroxy-iso-nonyl phthalate (MHiNP) was detected > LOQ in none of the samples, and therefore not displayed.

### Associations between endocrine disrupting chemicals and adiposity-related traits

**Figure 1:**
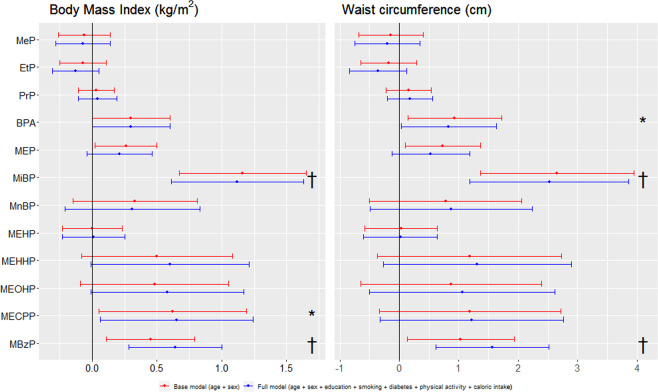
Multivariate associations between adiposity-related traits and urinary paraben, bisphenol and phthalate concentrations in the Lifelines population (n = 662). Data is presented as estimate [confidence interval] for two models. The base model (red) is corrected for age and sex. The full model (blue) is corrected for age, sex, education, smoking, diabetes status, physical activity and total caloric intake. Endocrine Disrupting Chemicals (EDCs) which were detected above the limit of quantification (LOQ) in at least 50% of the samples were included in analysis. EDCs were log_10_-transformed before analysis. For full names of EDCs, see Table 2. *raw *p*-value < 0.05 in full model; ^†^Benjamini and Hochberg False Discovery Rate (FDR < 0.05) in full model.

Figure 1 presents association analyses between urinary EDC concentrations and adiposity-related traits. The base model, which corrects for sex and age, showed direct associations for BPA, MEP, MiBP, MECPP and MBzP and BMI (all *p* < 0.05). When additionally correcting for level of education, smoking, diabetes, physical activity and caloric intake in the full model, the associations for BPA and MEP lost significance (*p* ≥ 0.05). Effect sizes for MiBP (Beta: 1.12, *p* < 0.0001) and MECPP (B: 0.65, *p* = 0.031) were robust to the additional adjustments, whereas the effect size for MBzP increased (B_base_:0.45, *p* = 0.0099; B_full_: 0.64, *p* = 0.0006). Waist circumference showed associations to similar EDCs, with direct associations for BPA, MEP, MiBP and MBzP in the base model. Of these associations, BPA (B: 0.83, *p* = 0.042) and MiBP (2.52, *p* = 0.0002) remained robust to additional adjustments in the full model. Like BMI, the effect size of MBzP increased in the full model (B_base_: 1.03, *p* = 0.0261; B_full_: 1.56, *p* = 0.001) The associations for MiBP and MBzP and both adiposity-related traits remained significant after adjusting for multiple testing (FDR:≤0.008). Estimates, confidence intervals, raw- and FDR-adjusted *p*-values are presented in Supplementary tables 2a,b.

### Associations between endocrine disrupting chemicals and other cardiometabolic traits

**Figure 2:**
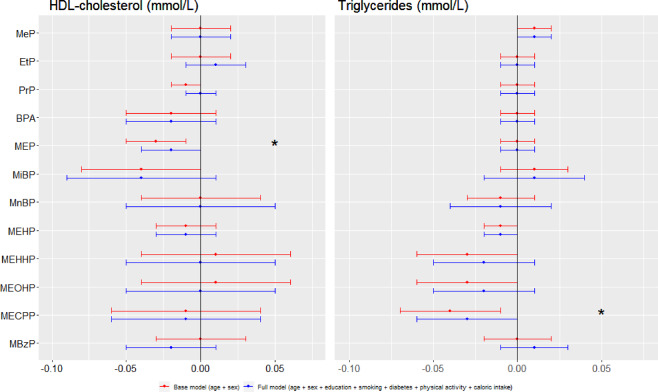
Multivariate associations between lipid-related traits and urinary paraben, bisphenol and phthalate concentrations in the Lifelines population (n = 662). Data is presented as estimate [confidence interval] for two models. The base model (red) is corrected for age and sex. The full model (blue) is corrected for age, sex, education, smoking, diabetes status, physical activity and total caloric intake. Endocrine Disrupting Chemicals (EDCs) which were detected above the limit of quantification (LOQ) in at least 50% of the samples were included in analysis. EDCs and triglycerides were log_10_-transformed before analysis. For full names of EDCs, see Table 2. *raw *p*-value < 0.05 in full model.

Associations between EDCs and lipids are presented in Fig. 2. When adjusting for age and sex, HDL-cholesterol showed an inverse relation with MEP (B: −0.03, *p* = 0.0168). Although this association weakened in the full model, it remained significant (B: −0.02, *p* = 0.0391). For triglycerides, we found inverse associations with MEHP (B: −0.01, *p* = 0.0106) and MECPP (B: −0.04, *p* = 0.0089). After adjusting for additional variables both effects weakened, resulting in the association with MEHP becoming non-significant and the one with MECPP reducing in effect size (−0.03, *p* = 0.0417). None of the associations between lipids and EDCs survived the correction for multiple testing (all FDR > 0.05). Glucose and blood pressure were not associated with any of the EDCs. Estimates, confidence intervals and *p*-values can be found in Supplementary Table 2c–g.

### Linearity of associations between endocrine disrupting chemicals and cardiometabolic traits

To evaluate if associations between continuous EDCs and cardiometabolic traits followed a linear relationship, EDCs were categorized into quartiles after which association analyses were repeated taking the first quartile (i.e. lowest exposure) as reference group. The results are presented in Supplementary Table 3a–d. For obesity-related traits, all significant associations originated from the third (versus first) or fourth (versus first) quartile. Although MiBP showed an association between its second versus first quartile, the effect sizes increased with higher quartiles confirming linearity (Beta’s; BMI: second: 1.03, third: 1.45; fourth versus first: 1.91; waist circumference: second: 2.76; third: 3.64; fourth versus first: 4.76). For lipids, significant associations originated solely from third or fourth EDC quartiles for all but MEHHP and triglycerides (second versus first quartile, B: 0.06, *p*-value: 0.0188). The second quartile versus first quartile of MnBP and EtP were associated for diastolic and systolic blood pressure, respectively. Although MiBP showed significant associations with systolic blood pressure for its highest quartiles, the association with the third quartile (versus first) was much stronger compared to that of the fourth quartile (versus first; B: 3.48, 2.72, respectively). No associations between EDCs and glucose were found in the quartile analysis.

## Discussion

In this study, exposure to five parabens, three bisphenols and thirteen metabolites of in total eight different phthalate diesters was assessed in 24 h urine samples of a general Dutch population. Next, we investigated potential associations between the EDCs which were quantified in at least 50% of the population and cardiometabolic traits, adjusting for risk factors including age, sex, education, smoking, diabetes status, physical activity and dietary intake.

Four parabens, BPA and more than half of the phthalate metabolites were detected in at least 90% of the 24 h urine samples, suggesting a ubiquitous exposure to these compounds in a general population from Northern Netherlands. To date, only two studies have examined a similar set of EDCs in the Netherlands, using a population of pregnant women from the Generation R study^51,52^. The most recent study by Philips *et al*. showed similar (i.e. <15% difference between medians) concentrations for BPA (median [25^th^; 75^th^ quartile]: 1.90 [0.96; 3.63] vs 1.66 [0.72; 3.56] ng/mL), MiBP (19.7 [12.1; 34.2] vs 21.6 [9.55; 45.9] ng/mL), MnBP (17.5 [10.8; 29.0] vs 16.2 [7.01; 31.2] ng/mL), and MBzP (5.84 [3.27; 11.0] vs 6.59 [3.07; 12.9] ng/mL). However, in current study we detected lower concentrations of BPF (0.24 [<LOD; 0.68] vs 0.57 [0.30; 1.29 ng/mL), MMP (0.48 [<LOD; 1.20] vs 5.43 [2.75; 9.88] ng/mL), MEP (47.1 [20.0; 132] vs 138 [41.2; 487 ng/mL), and all DEHP metabolites (9.12 [6.20; 14.0] vs 12.0 [5.83; 23.2]; 6.15 [4.10; 9.60] vs 7.81 [3.53; 15.5]; 10.3 [6.60; 16.0] vs 16.4 [8.26; 31.8] ng/mL, MEHHP, MEOHP, MECPP, respectively). The observed discrepancies could be due to several reasons. First, the study population differed (i.e. general population vs pregnant women). Second, urine samples from the Generation R study were collected in 2004–2005, whereas samples from the current study were collected in 2012. During this time, EDCs used in consumer products could have changed, partly due to a rise in public awareness of the potential health effects of EDCs. A study investigating temporal trends of phthalates over similar time period (2001–2010) in NHANES showed a decrease urinary phthalate concentrations similar to our findings^53^. Third, Philips *et al*. determined EDC concentrations in spot urine. Although Christensen *et al*. showed spot urine samples to be roughly comparable with 24 h urine samples, several studies detected higher EDC concentrations in spot urine compared to 24 h urine^54–56^.

In general, we detected EDCs concentrations in the same ranges as other European countries^19,57–64^, although there are differences and variation in reported levels between studies and countries. Also, the Center of Disease and Control (CDC) reported largely similar paraben, BPA and phthalate levels in the United States of America^18^.

The direct and inverse associations between parabens and adiposity-related traits which we observed in this study did not reach significance. A recent study by Kolatorova *et al*. showed higher concentrations of MeP and PrP in serum of obese compared to normal-weight individuals^65^, supporting the findings in a large Korean cohort^66^. Yet, a large cross-sectional study including 4,730 adults from NHANES found inverse associations between parabens and adiposity^36^. Therefore, research is needed in prospective populations to further investigate the effects of parabens on adiposity.

A large body of evidence showed the obesogenic properties of BPA and phthalates^67^. In this study, we found associations between BPA and several phthalate metabolites (i.e. DEHP, MiBP, and MBzP) and adiposity related traits. Positive estimates for BPA and DEHP did not hold up after correction for multiple testing in the current study. However, other studies with larger sample sizes than the current study did report significant effects^6,68^. Associations found for MiBP and MBzP were in line with cross-sectional and prospective studies^44,69–71^.

Previously, BPA and phthalates have been linked to both impaired glucose metabolism^6,11,37,72,73^, as well as T2D^26,47,73–75^. In this study, we did not find any significant associations between EDCs and glycaemic traits. This could be explained by the observational design of this study in a healthy population (diabetic patients were excluded from glycaemic trait analysis) and insufficient statistical power to detect minor differences in a small range of glucose.

Evidence regarding associations between EDCs and lipid traits have been mixed. We detected an inverse association between MEHP and triglycerides, which did not remain significant after adjusting for multiple testing. Although Dong *et al*. showed higher urinary levels of MMP to be associated with hyperlipidemia, they also found an inverse association between MEHP and hyperlipidemia in line with our findings^34^. In contrast, James-Todd *et al*. found DEHP to be associated with hypertriglyceridemia^6^.

The relationship of EDCs with blood pressure traits remains unclear. Previously, several studies that investigated associations between EDCs and blood pressure in pregnant women found inverse associations for parabens but direct associations with MBzP^33,35^. In a population of elderly people, MEP was inversely associated with diastolic blood pressure^37^. We did not find any significant associations between EDCs and blood pressure.

Like natural hormones, EDCs have been shown to follow nonmonotonic dose-response curves^48,49^. When we tested associations between continuous EDCs and cardiometabolic traits in our main analysis, linearity was assumed. As it is possible that associations have non-linear effects, we assessed such potential non-linear associations by categorizing the EDCs of interest into quartiles. For all significant associations reported in the main analysis (using continuous EDCs), non-linear associations were not observed. However, some signs of non-linearity were found for blood pressure. Therefore, the findings for continuous EDCs and blood pressure should be interpreted with care.

The strengths of the current study include its population-based design, combined with objective anthropometric measurements performed by a trained research nurse, extensive data on dietary intake and physical activity and measurements of EDC exposure in 24 h urine samples.

The Lifelines adult study population is broadly representative for the north of the Netherlands^39^ and the characteristics of the current study population are comparable with the Dutch general population (i.e., similar age, sex-ratio and percentage of subjects with overweight; 45.8 versus 40.1 years; males: 42 versus 42%; overweight: 47 versus 48%, respectively)^76^. This implies that our data reflect the EDC exposure in the Netherlands.

Due to the extensive data collected in the Lifelines cohort, we were able to adjust for confounding factors identified in earlier studies (i.e. age, sex, education, smoking, type 2 diabetes), as well as include traditional risk factors known to have adverse effects on cardiometabolic traits. Variables such as physical activity and caloric intake have a big impact on the cardiometabolic traits but are often not considered when investigating associations with EDCs. By including these lifestyle factors in the current study, we were able to test associations between EDCs and cardiometabolic traits while taking these classical risk factors into account.

In current study, EDCs levels were measured in urine collected over 24 h. It has been shown that parabens, BPA and phthalates are largely excreted in urine within 24 h after oral administration^14–16^. Moreover, urinary concentrations of parabens and phthalates increased in a few hours after topical application^77^. Due to this fast metabolism and excretion, 24 h urine samples provide a reliable estimate of exposure. While EDCs can also be measured in other biospecimens such as blood serum, adipose tissue and brain tissue^78–81^, its collection requires invasive methods and is time-consuming and expensive. Therefore, urine is the most suitable medium for the determination of these EDCs in large population studies.

As 24 h urine is relatively strenuous to obtain and requires motivated participants, many studies use spot- or morning-urine as proxy for chronic EDC exposure. In order to adjust these urinary EDC concentrations for dilution different techniques are used, mostly correcting for creatinine. Yet, creatinine levels vary by sex, age, race, diet, and activity^82^. Therefore, different techniques have shown to introduce inconsistencies in association analyses^83^. By using 24 h urine, urine dilution can be accounted for by multiplying raw EDC concentrations by the total volume of excreted urine to calculate urinary EDC excretion in mL/24 h. Therefore, it is expressed as an excretion rate (i.e. per-volume basis) and does not need additional adjustments^82,84^. Finally, the EDC concentrations were quantified by LC-MS/MS presently regarded as the gold standard technique, providing accurate data on exposures.

Several studies include a multitude of chemicals, which are often limited to a single compound group (i.e. or parabens, or bisphenols, or phthalates). By using two LC-MS/MS methods, we were able to measure a broad set of EDC concentrations at the same time, covering the most common groups of non-persistent endocrine disruptors to which we are exposed in our daily life.

As this study is cross-sectional, results should be interpreted with caution. The direction of the associations found between EDCs and adiposity-related traits cannot be determined in the current design. Consequently, these data need to be confirmed in prospective studies. Our study population consisted of 662 subjects. Although this is a relatively big sample size when taking the collection of 24 h urine into account, associations between EDCs and cardiometabolic traits are often subtle, requiring a large power. In this study, many associations were found to be too weak to withstand correction for multiple testing. Therefore, larger studies are needed in the future.

## Conclusions

In conclusion, we for the first-time assessed the exposure to the most common environmental chemicals such as parabens, bisphenols and phthalates in the Dutch population and evaluated its association with cardiometabolic profiles. Our data suggest obesogenic properties of some phthalates. These findings support the potency of EDCs to have clinically relevant effects on cardiometabolic health. Further research is warranted to expand our understanding of the impact of environmental chemicals on human health.

## Supplementary information

Supplementary tables.
