# Supplementary material for: Exposure to Endocrine Disrupting Chemicals in the Dutch general population is associated with adiposity-related traits
Source: Sci Rep. 2020 Jun 9;10:9311. doi: 10.1038/s41598-020-66284-3 (PMC7283255; doi:10.1038/s41598-020-66284-3)
Supplement: Supplementary file 1 — Supplementary tables. [file 41598_2020_66284_MOESM1_ESM.docx]

**Exposure to Endocrine Disrupting Chemicals in the Dutch general population is associated with adiposity-related traits**

Thomas P. van der Meer, Martijn van Faassen, André P. van Beek, Harold Snieder, Ido P. Kema, Bruce H.R. Wolffenbuttel, Jana V. van Vliet-Ostaptchouk

**Supplementary table 1a**. Cone voltage and collision energies for parabens and phenols.

| Compound | Abbreviation | Abbreviation (setting) | Precursor m/z | Product m/z | Cone voltage (V) | Collision energy (eV) |
| --- | --- | --- | --- | --- | --- | --- |
| Methyl paraben | MeP | MeP-1 (QN) | 151.05 | 92.00 | 30 | 19 |
|  |  | MeP-2 (QL) | 151.05 | 136.00 | 30 | 13 |
|  |  | MeP-IS (QN) | 157.20 | 98.10 | 30 | 19 |
|  |  | MeP-IS | 157.20 | 142.10 | 30 | 13 |
| Ethyl paraben | EtP | EtP (QN) | 165.17 | 92.00 | 30 | 20 |
|  |  | EtP-IS (QN) | 169.20 | 96.10 | 30 | 20 |
| Propyl paraben | PrP | PrP(QN) | 179.00 | 92.00 | 30 | 23 |
|  |  | PrP-IS (QN) | 186.20 | 92.10 | 30 | 23 |
| n-Butyl paraben | n-BuP | n-BuP (QN) | 193.10 | 92.00 | 30 | 28 |
|  |  | n-BuP-IS (QN) | 199.20 | 98.10 | 30 | 28 |
| Benzyl paraben | BzP | BzP (QN) | 227.15 | 92.05 | 30 | 35 |
|  |  | BzP-IS1 (QN) | 234.15 | 92.10 | 30 | 35 |
| Bisphenol A | BPA | BP-A (QN) | 227.15 | 133.05 | 30 | 26 |
|  |  | BP-A-IS (QN) | 239.20 | 139.15 | 30 | 25 |
| Bisphenol F | BPF | BP-F (QN) | 199.10 | 93.00 | 30 | 18 |
|  |  | BP-F-IS (QN) | 205.00 | 99.00 | 30 | 18 |
| Bisphenol S | BPS | BP-S (QN) | 249.05 | 92.00 | 30 | 40 |
|  |  | BP-IS (QN) | 261.20 | 98.10 | 30 | 40 |

QN, quantitative; QL, qualitative

**Supplementary table 1b.** Cone voltage and collision energies for phthalate metabolites.

| Compound | Abbreviation | Abbreviation  (setting) | Precursor (m/z) | Product (m/z) | Cone Voltage (V) | Collision energy (V) |
| --- | --- | --- | --- | --- | --- | --- |
| Mono-n-pentyl phthalate | MMP | MMP1 (QN) | 179.05 | 77 | 30 | 17 |
|  |  | MMP2 (QL) | 179.05 | 107 | 30 | 10 |
|  |  | MMP-13C | 183.0 | 79 | 30 | 17 |
| Mono-ethyl phthalate | MEP | MEP1 (QL) | 193 | 121 | 30 | 11 |
|  |  | MEP2 (QN) | 193 | 77 | 30 | 15 |
|  |  | MEP-13C | 197.1 | 79 | 30 | 15 |
| Mono-iso-butyl phthalate | MiBP | MiBP2- | 221.05 | 77 | 30 | 16 |
|  |  | MiBP3 (QL) | 221.05 | 134 | 30 | 14 |
|  |  | MiBP-d4 | 225.05 | 71 | 30 | 14 |
| Mono-n-butyl phthalate | MnBP | MnBP1 (QL) | 221.05 | 71 | 30 | 14 |
|  |  | MnBP2 (QN) |  |  |  |  |
|  |  | MnBP-13C | 225.05 | 79 | 30 | 16 |
| Mono-(2-ethylhexyl) phthalate | MEHP | MEHP1 (QL) | 277.15 | 77 | 30 | 19 |
|  |  | MEHP3 (QN) | 277.15 | 134 | 30 | 15 |
|  |  | MEHP-13C | 281.15 | 137 | 30 | 15 |
| Mono-n-hexyl phthalate | MnHP | MnHP1 (QN) | 249.07 | 77 | 40 | 18 |
|  |  | MnHP2 (QL) | 249.07 | 99 | 40 | 15 |
|  |  | MnHP-d4 | 253.07 | 81 | 40 | 18 |
| Mono-(2-ethyl-5-hydroxyhexyl) phthalate | MEHHP | MEHHP2 (QL) | 293.13 | 121 | 30 | 20 |
|  |  | MEHHP3 (QN) | 293.13 | 145 | 30 | 14 |
|  |  | MEHHP-13C | 297.13 | 124 | 30 | 20 |
| Mono-(2-ethyl-5-oxohexyl) phthalate | MEOHP | MEOHP2 (QN) | 291.13 | 121 | 30 | 19 |
|  |  | MEOHP3 (QL) | 291.13 | 143 | 30 | 14 |
|  |  | MEOHP-13C | 295.13 | 124 | 30 | 14 |
| Mono-(2-ethyl-5-carboxypentyl) phthalate | MECPP | MECPP2 (QL) | 307.2 | 113 | 35 | 28 |
|  |  | MECPP3 (QN) | 307.2 | 159 | 35 | 11 |
|  |  | MECPP-13C | 311.25 | 159 | 35 | 11 |
| Mono-benzyl phthalate | MBzP | MBzP2 (QN) | 255.05 | 107 | 30 | 14 |
|  |  | MBzp3 (QL) | 255.05 | 183 | 30 | 11 |
|  |  | MBzP-13C | 259.05 | 186 | 30 | 14 |
| Mono-iso-nonyl phthalate | MiNP | MiNP1 (QL) | 291.15 | 77 | 30 | 22 |
|  |  | MiNP2 (QN) | 291.15 | 141 | 30 | 19 |
|  |  | MiNP-13C | 295.25 | 79 | 30 | 24 |
| Mono-hydroxy-iso-nonyl phthalate | MHiNP |  |  |  |  |  |
|  |  |  |  |  |  |  |
| Mono-iso-decyl phthalate | MiDP | MiDP1 (QN) | 305.15 | 77 | 30 | 21 |
|  |  | MiDP3 (QL) | 305.15 | 155 | 30 | 17 |

QN, quantitative; QL, qualitative

**Supplementary table 2a.** Multivariate associations between Body Mass Index and urinary paraben, bisphenol and phthalate concentrations in the Lifelines population (n = 662).

|  | Base model | | | Full model | | |
| --- | --- | --- | --- | --- | --- | --- |
|  | Estimate [CI] | p-value | FDR | Estimate [CI] | p-value | FDR |
| MeP | -0.06 [-0.26, 0.14] | 0.5423 | 0.6507 | -0.07 [-0.28, 0.14] | 0.4949 | 0.5938 |
| EtP | -0.07 [-0.25, 0.11] | 0.4357 | 0.5809 | -0.13 [-0.31, 0.05] | 0.1553 | 0.2329 |
| PrP | 0.03 [-0.11, 0.17] | 0.6457 | 0.7044 | 0.04 [-0.11, 0.19] | 0.6111 | 0.6666 |
| BPA | 0.30 [0.00, 0.60] | **0.0467** | 0.1120 | 0.30 [0.00, 0.60] | 0.0506 | 0.1092 |
| MEP | 0.26 [0.02, 0.50] | **0.0316** | 0.0999 | 0.21 [-0.04, 0.46] | 0.0930 | 0.1594 |
| MiBP | 1.16 [0.67, 1.65] | **<0.0001** | **<0.0001** | 1.12 [0.61, 1.63] | **<0.0001** | **0.0002** |
| MnBP | 0.33 [-0.15, 0.81] | 0.1785 | 0.2678 | 0.31 [-0.21, 0.83] | 0.2409 | 0.3212 |
| MEHP | 0.00 [-0.23, 0.23] | 0.9970 | 0.9970 | 0.01 [-0.23, 0.25] | 0.9191 | 0.9191 |
| MEHHP | 0.50 [-0.08, 1.08] | 0.0936 | 0.1740 | 0.60 [-0.01, 1.21] | 0.0541 | 0.1092 |
| MEOHP | 0.48 [-0.09, 1.05] | 0.1015 | 0.1740 | 0.58 [-0.01, 1.17] | 0.0546 | 0.1092 |
| MECPP | 0.62 [0.05, 1.19] | **0.0333** | 0.0999 | 0.65 [0.06, 1.24] | **0.0309** | 0.1092 |
| MBzP | 0.45 [0.11, 0.79] | **0.0099** | 0.0596 | 0.64 [0.28, 1.00] | **0.0006** | **0.0036** |

CI, Confidence Interval; FDR, False Discovery Rate. The base model was corrected for age and sex. The full model was corrected for age, sex, education, smoking, diabetes status, physical activity and total caloric intake. Endocrine Disrupting Chemicals (EDCs) which were detected above the limit of quantification (LOQ) in at least 50% of the samples were included in analysis. EDCs were log10-transformed to adjust for right-skewed distribution. For full names of EDCs, see supplementary table 1a and 1b. Raw *p*-values <0.05 and Benjamini and Hochberg FDR < 0.05 are expressed bold.

|  | Base model | | | Full model | | |
| --- | --- | --- | --- | --- | --- | --- |
|  | Estimate [CI] | p-value | FDR | Estimate [CI] | p-value | FDR |
| MeP | -0.14 [-0.68, 0.40] | 0.6119 | 0.6675 | -0.20 [-0.75, 0.35] | 0.4710 | 0.5138 |
| EtP | -0.18 [-0.65, 0.29] | 0.4608 | 0.5529 | -0.36 [-0.84, 0.12] | 0.1433 | 0.2456 |
| PrP | 0.16 [-0.22, 0.54] | 0.3990 | 0.5320 | 0.18 [-0.20, 0.56] | 0.3557 | 0.4268 |
| BPA | 0.93 [0.14, 1.72] | **0.0207** | 0.0783 | 0.83 [0.03, 1.63] | **0.0420** | 0.1681 |
| MEP | 0.73 [0.10, 1.36] | **0.0232** | 0.0783 | 0.53 [-0.12, 1.18] | 0.1124 | 0.2456 |
| MiBP | 2.65 [1.36, 3.94] | **0.0001** | **0.0008** | 2.52 [1.19, 3.85] | **0.0002** | **0.0028** |
| MnBP | 0.78 [-0.50, 2.06] | 0.2338 | 0.3954 | 0.87 [-0.49, 2.23] | 0.2117 | 0.2822 |
| MEHP | 0.03 [-0.58, 0.64] | 0.9174 | 0.9174 | 0.02 [-0.60, 0.64] | 0.9379 | 0.9379 |
| MEHHP | 1.18 [-0.37, 2.73] | 0.1357 | 0.2713 | 1.31 [-0.27, 2.89] | 0.1043 | 0.2456 |
| MEOHP | 0.87 [-0.65, 2.39] | 0.2636 | 0.3954 | 1.06 [-0.50, 2.62] | 0.1818 | 0.2726 |
| MECPP | 1.19 [-0.33, 2.71] | 0.1239 | 0.2713 | 1.22 [-0.32, 2.76] | 0.1228 | 0.2456 |
| MBzP | 1.03 [0.13, 1.93] | **0.0261** | 0.0783 | 1.56 [0.61, 2.51] | **0.0013** | **0.0078** |

**Supplementary table 2b.** Multivariate associations between waist circumference and urinary paraben, bisphenol and phthalate concentrations in the Lifelines population (n = 662).

CI, Confidence Interval; FDR, False Discovery Rate. The base model was corrected for age and sex. The full model was corrected for age, sex, education, smoking, diabetes status, physical activity and total caloric intake. Endocrine Disrupting Chemicals (EDCs) which were detected above the limit of quantification (LOQ) in at least 50% of the samples were included in analysis. EDCs were log10-transformed to adjust for right-skewed distribution. For full names of EDCs, see supplementary table 1a and 1b. Raw *p*-values <0.05 and Benjamini and Hochberg FDR < 0.05 are expressed bold.

|  | Base model | | | Full model | | |
| --- | --- | --- | --- | --- | --- | --- |
|  | Estimate [CI] | p-value | FDR | Estimate [CI] | p-value | FDR |
| MeP | 0.00 [-0.02, 0.02] | 0.7915 | 0.8490 | 0.00 [-0.02, 0.02] | 0.8933 | 0.9683 |
| EtP | 0.00 [-0.02, 0.02] | 0.7021 | 0.8490 | 0.01 [-0.01, 0.03] | 0.1977 | 0.5692 |
| PrP | -0.01 [-0.02, 0.00] | 0.3904 | 0.8490 | 0.00 [-0.01, 0.01] | 0.4475 | 0.8950 |
| BPA | -0.02 [-0.05, 0.01] | 0.1173 | 0.4691 | -0.02 [-0.05, 0.01] | 0.1353 | 0.5410 |
| MEP | -0.03 [-0.05, -0.01] | **0.0168** | 0.2021 | -0.02 [-0.04, 0.00] | **0.0391** | 0.4289 |
| MiBP | -0.04 [-0.08, 0.00] | 0.0906 | 0.4691 | -0.04 [-0.09, 0.01] | 0.0715 | 0.4289 |
| MnBP | 0.00 [-0.04, 0.04] | 0.8490 | 0.8490 | 0.00 [-0.05, 0.05] | 0.9281 | 0.9683 |
| MEHP | -0.01 [-0.03, 0.01] | 0.5602 | 0.8490 | -0.01 [-0.03, 0.01] | 0.5478 | 0.9392 |
| MEHHP | 0.01 [-0.04, 0.06] | 0.7690 | 0.8490 | 0.00 [-0.05, 0.05] | 0.9683 | 0.9683 |
| MEOHP | 0.01 [-0.04, 0.06] | 0.5981 | 0.8490 | 0.00 [-0.05, 0.05] | 0.8575 | 0.9683 |
| MECPP | -0.01 [-0.06, 0.04] | 0.7891 | 0.8490 | -0.01 [-0.06, 0.04] | 0.6574 | 0.9683 |
| MBzP | 0.00 [-0.03, 0.03] | 0.8442 | 0.8490 | -0.02 [-0.05, 0.01] | 0.2372 | 0.5692 |

**Supplementary table 2c.** Multivariate associations between HDL-cholesterol and urinary paraben, bisphenol and phthalate concentrations in the Lifelines population (n = 662).

CI, Confidence Interval; FDR, False Discovery Rate. The base model was corrected for age and sex. The full model was corrected for age, sex, education, smoking, diabetes status, physical activity and total caloric intake. Endocrine Disrupting Chemicals (EDCs) which were detected above the limit of quantification (LOQ) in at least 50% of the samples were included in analysis. EDCs were log10-transformed to adjust for right-skewed distribution. For full names of EDCs, see supplementary table 1a and 1b. Raw *p*-values <0.05 and Benjamini and Hochberg FDR < 0.05 are expressed bold.

|  | Base model | | | Full model | | |
| --- | --- | --- | --- | --- | --- | --- |
|  | Estimate [CI] | p-value | FDR | Estimate [CI] | p-value | FDR |
| MeP | 0.01 [0.00, 0.02] | 0.1412 | 0.3389 | 0.01 [0.00, 0.02] | 0.1458 | 0.4375 |
| EtP | 0.00 [-0.01, 0.01] | 0.2694 | 0.4618 | 0.00 [-0.01, 0.01] | 0.3894 | 0.6675 |
| PrP | 0.00 [-0.01, 0.01] | 0.2013 | 0.4026 | 0.00 [-0.01, 0.01] | 0.1938 | 0.4652 |
| BPA | 0.00 [-0.01, 0.01] | 0.9600 | 0.9600 | 0.00 [-0.01, 0.01] | 0.9863 | 0.9863 |
| MEP | 0.00 [-0.01, 0.01] | 0.9505 | 0.9600 | 0.00 [-0.01, 0.01] | 0.7869 | 0.8585 |
| MiBP | 0.01 [-0.01, 0.03] | 0.4608 | 0.6145 | 0.01 [-0.02, 0.04] | 0.4847 | 0.6848 |
| MnBP | -0.01 [-0.03, 0.01] | 0.3662 | 0.5493 | -0.01 [-0.04, 0.02] | 0.5777 | 0.6932 |
| MEHP | -0.01 [-0.02, 0.00] | **0.0106** | 0.0635 | -0.01 [-0.02, 0.00] | 0.0538 | 0.3230 |
| MEHHP | -0.03 [-0.06, 0.00] | 0.0777 | 0.2332 | -0.02 [-0.05, 0.01] | 0.2815 | 0.5631 |
| MEOHP | -0.03 [-0.06, 0.00] | **0.0173** | 0.0690 | -0.02 [-0.05, 0.01] | 0.1094 | 0.4375 |
| MECPP | -0.04 [-0.07, -0.01] | **0.0089** | 0.0635 | -0.03 [-0.06, 0.00] | **0.0417** | 0.3230 |
| MBzP | 0.00 [-0.02, 0.02] | 0.7711 | 0.9253 | 0.01 [-0.01, 0.03] | 0.5136 | 0.6848 |

**Supplementary table 2d.** Multivariate associations between triglycerides and urinary paraben, bisphenol and phthalate concentrations in the Lifelines population (n = 662).

CI, Confidence Interval; FDR, False Discovery Rate. The base model was corrected for age and sex. The full model was corrected for age, sex, education, smoking, diabetes status, physical activity and total caloric intake. Endocrine Disrupting Chemicals (EDCs) which were detected above the limit of quantification (LOQ) in at least 50% of the samples were included in analysis. EDCs were log10-transformed to adjust for right-skewed distribution. For full names of EDCs, see supplementary table 1a and 1b. Raw *p*-values <0.05 and Benjamini and Hochberg FDR < 0.05 are expressed bold.

**Supplementary table 2e.** Multivariate associations between fasting glucose and urinary paraben, bisphenol and phthalate concentrations in the Lifelines population (n = 662).

|  | Base model | | | Full model | | |
| --- | --- | --- | --- | --- | --- | --- |
|  | Estimate [CI] | p-value | FDR | Estimate [CI] | p-value | FDR |
| MeP | 0.00 [-0.02, 0.02] | 0.7674 | 0.9994 | 0.00 [-0.02, 0.02] | 0.8062 | 0.9892 |
| EtP | -0.01 [-0.03, 0.01] | 0.4745 | 0.9994 | -0.01 [-0.03, 0.01] | 0.5349 | 0.9892 |
| PrP | 0.01 [0.00, 0.02] | 0.0512 | 0.6143 | 0.01 [-0.01, 0.03] | 0.0653 | 0.7831 |
| BPA | 0.00 [-0.03, 0.03] | 0.8478 | 0.9994 | 0.01 [-0.02, 0.04] | 0.6978 | 0.9892 |
| MEP | 0.01 [-0.01, 0.03] | 0.6144 | 0.9994 | 0.00 [-0.03, 0.03] | 0.9552 | 0.9892 |
| MiBP | 0.02 [-0.03, 0.07] | 0.5583 | 0.9994 | 0.00 [-0.05, 0.05] | 0.9023 | 0.9892 |
| MnBP | 0.01 [-0.04, 0.06] | 0.5855 | 0.9994 | 0.00 [-0.05, 0.05] | 0.9892 | 0.9892 |
| MEHP | -0.02 [-0.04, 0.00] | 0.1878 | 0.9994 | -0.01 [-0.03, 0.01] | 0.2469 | 0.9892 |
| MEHHP | 0.00 [-0.06, 0.06] | 0.9994 | 0.9994 | 0.01 [-0.05, 0.07] | 0.6596 | 0.9892 |
| MEOHP | 0.00 [-0.06, 0.06] | 0.9973 | 0.9994 | 0.01 [-0.05, 0.07] | 0.6644 | 0.9892 |
| MECPP | 0.01 [-0.05, 0.07] | 0.7300 | 0.9994 | 0.02 [-0.04, 0.08] | 0.6005 | 0.9892 |
| MBzP | 0.02 [-0.01, 0.05] | 0.3688 | 0.9994 | 0.02 [-0.02, 0.06] | 0.2895 | 0.9892 |

CI, Confidence Interval; FDR, False Discovery Rate. The base model was corrected for age and sex. The full model was corrected for age, sex, socio-economic status, smoking, physical activity and total caloric intake. Endocrine Disrupting Chemicals (EDCs) which were detected above the limit of quantification (LOQ) in at least 50% of the samples were included in analysis. EDCs were log10-transformed to adjust for right-skewed distribution. All individuals which were diagnosed with diabetes (n = 8), or which had fasting glucose levels ≥ 7.0 mmol/L (n = 41) were excluded from analysis. For full names of EDCs, see supplementary table 1a and 1b. Raw *p*-values <0.05 and Benjamini and Hochberg FDR < 0.05 are expressed bold.

|  | Base model | | | Full model | | |
| --- | --- | --- | --- | --- | --- | --- |
|  | Estimate [CI] | p-value | FDR | Estimate [CI] | p-value | FDR |
| MeP | 0.03 [-0.38, 0.44] | 0.8982 | 0.9678 | 0.08 [-0.35, 0.51] | 0.7182 | 0.9231 |
| EtP | -0.01 [-0.36, 0.34] | 0.9678 | 0.9678 | -0.07 [-0.44, 0.30] | 0.7173 | 0.9231 |
| PrP | 0.07 [-0.21, 0.35] | 0.6261 | 0.9678 | 0.07 [-0.23, 0.37] | 0.6386 | 0.9231 |
| BPA | -0.17 [-0.77, 0.43] | 0.5846 | 0.9678 | -0.23 [-0.85, 0.39] | 0.4637 | 0.9231 |
| MEP | -0.16 [-0.64, 0.32] | 0.5139 | 0.9678 | -0.08 [-0.58, 0.42] | 0.7458 | 0.9231 |
| MiBP | -0.04 [-1.03, 0.95] | 0.9408 | 0.9678 | 0.05 [-0.99, 1.09] | 0.9185 | 0.9625 |
| MnBP | 0.04 [-0.93, 1.01] | 0.9337 | 0.9678 | 0.24 [-0.81, 1.29] | 0.6536 | 0.9231 |
| MEHP | 0.01 [-0.45, 0.47] | 0.9565 | 0.9678 | 0.01 [-0.47, 0.49] | 0.9625 | 0.9625 |
| MEHHP | -0.26 [-1.43, 0.91] | 0.6643 | 0.9678 | -0.18 [-1.4, 1.04] | 0.7693 | 0.9231 |
| MEOHP | -0.38 [-1.53, 0.77] | 0.5128 | 0.9678 | -0.19 [-1.39, 1.01] | 0.7611 | 0.9231 |
| MECPP | -0.51 [-1.66, 0.64] | 0.3822 | 0.9678 | -0.43 [-1.62, 0.76] | 0.4847 | 0.9231 |
| MBzP | -0.40 [-1.09, 0.29] | 0.2506 | 0.9678 | -0.23 [-0.97, 0.51] | 0.5409 | 0.9231 |

**Supplementary table 2f.** Multivariate associations between diastolic blood pressure and urinary paraben, bisphenol and phthalate concentrations in the Lifelines population (n = 662).

CI, Confidence Interval; FDR, False Discovery Rate. The base model was corrected for age and sex. The full model was corrected for age, sex, education, smoking, diabetes status, physical activity and total caloric intake. Endocrine Disrupting Chemicals (EDCs) which were detected above the limit of quantification (LOQ) in at least 50% of the samples were included in analysis. EDCs were log10-transformed to adjust for right-skewed distribution. For full names of EDCs, see supplementary table 1a and 1b. Raw *p*-values <0.05 and Benjamini and Hochberg FDR < 0.05 are expressed bold.

|  | Base model | | | Full model | | |
| --- | --- | --- | --- | --- | --- | --- |
|  | Estimate [CI] | p-value | FDR | Estimate [CI] | p-value | FDR |
| MeP | -0.04 [-0.62, 0.54] | 0.8957 | 0.9508 | -0.07 [-0.67, 0.53] | 0.8104 | 0.8841 |
| EtP | -0.11 [-0.61, 0.39] | 0.6601 | 0.9069 | -0.28 [-0.80, 0.24] | 0.2927 | 0.8725 |
| PrP | -0.02 [-0.42, 0.38] | 0.9242 | 0.9508 | 0.00 [-0.41, 0.41] | 0.9947 | 0.9947 |
| BPA | 0.03 [-0.82, 0.88] | 0.9508 | 0.9508 | -0.18 [-1.04, 0.68] | 0.6799 | 0.8725 |
| MEP | 0.14 [-0.54, 0.82] | 0.6802 | 0.9069 | 0.26 [-0.44, 0.96] | 0.4653 | 0.8725 |
| MiBP | 0.72 [-0.69, 2.13] | 0.3197 | 0.9069 | 0.85 [-0.61, 2.31] | 0.2552 | 0.8725 |
| MnBP | 0.44 [-0.94, 1.82] | 0.5348 | 0.9069 | 0.55 [-0.92, 2.02] | 0.4628 | 0.8725 |
| MEHP | 0.14 [-0.52, 0.80] | 0.6696 | 0.9069 | 0.20 [-0.47, 0.87] | 0.5621 | 0.8725 |
| MEHHP | -0.66 [-2.33, 1.01] | 0.4405 | 0.9069 | -0.31 [-2.02, 1.4] | 0.7271 | 0.8725 |
| MEOHP | -0.8 [-2.43, 0.83] | 0.3346 | 0.9069 | -0.34 [-2.02, 1.34] | 0.6908 | 0.8725 |
| MECPP | -1.05 [-2.68, 0.58] | 0.2080 | 0.9069 | -0.87 [-2.54, 0.80] | 0.3079 | 0.8725 |
| MBzP | 0.23 [-0.75, 1.21] | 0.6384 | 0.9069 | 0.61 [-0.42, 1.64] | 0.2472 | 0.8725 |

**Supplementary table 2g.** Multivariate associations between systolic blood pressure and urinary paraben, bisphenol and phthalate concentrations in the Lifelines population (n = 662).

CI, Confidence Interval; FDR, False Discovery Rate. The base model was corrected for age and sex. The full model was corrected for age, sex, education, smoking, diabetes status, physical activity and total caloric intake. Endocrine Disrupting Chemicals (EDCs) which were detected above the limit of quantification (LOQ) in at least 50% of the samples were included in analysis. EDCs were log10-transformed to adjust for right-skewed distribution. For full names of EDCs, see supplementary table 1a and 1b. Raw *p*-values <0.05 and Benjamini and Hochberg FDR < 0.05 are expressed bold.

**Supplementary table 3a.** Multivariate associations between adiposity-related traits and urinary paraben, bisphenol and phthalate concentrations expressed as quartiles in the Lifelines population.

|  |  | Body Mass Index (kg/m^2^) | | Waist circumference (cm) | |
| --- | --- | --- | --- | --- | --- |
|  | Quartile | Estimate [CI] | *p*-value | Estimate [CI] | *p*-value |
| MeP | 2 | -0.13 [-1.07, 0.81] | 0.7916 | -0.15 [-2.60, 2.30] | 0.9050 |
|  | 3 | -0.73 [-1.67, 0.21] | 0.1314 | -1.74 [-4.20, 0.72] | 0.1678 |
|  | 4 | -0.10 [-1.06, 0.86] | 0.8316 | -0.10 [-2.62, 2.42] | 0.9378 |
| EtP | 2 | -0.21 [-1.15, 0.73] | 0.6647 | -1.05 [-3.51, 1.41] | 0.4049 |
|  | 3 | -0.02 [-0.97, 0.93] | 0.9632 | 0.21 [-2.26, 2.68] | 0.8699 |
|  | 4 | -0.65 [-1.61, 0.31] | 0.1802 | -1.94 [-4.43, 0.55] | 0.1286 |
| PrP | 2 | 0.06 [-0.88, 1.00] | 0.8968 | 0.61 [-1.85, 3.07] | 0.6251 |
|  | 3 | 0.03 [-0.92, 0.98] | 0.9503 | -0.39 [-2.87, 2.09] | 0.7609 |
|  | 4 | 0.27 [-0.68, 1.22] | 0.5788 | 1.43 [-1.05, 3.91] | 0.2583 |
| BPA | 2 | 0.04 [-0.91, 0.99] | 0.9381 | 0.79 [-1.69, 3.27] | 0.5304 |
|  | 3 | 0.70 [-0.24, 1.64] | 0.1451 | 2.95 [0.50, 5.40] | **0.0190** |
|  | 4 | 0.93 [0.00, 1.86] | 0.0525 | 2.34 [-0.10, 4.78] | 0.0604 |
| MEP | 2 | 0.89 [-0.05, 1.83] | 0.0631 | 2.03 [-0.42, 4.48] | 0.1050 |
|  | 3 | 0.13 [-0.82, 1.08] | 0.7865 | 0.41 [-2.08, 2.90] | 0.7476 |
|  | 4 | 0.85 [-0.10, 1.8] | 0.0785 | 2.24 [-0.23, 4.71] | 0.0761 |
| MiBP | 2 | 1.03 [0.09, 1.97] | **0.0315** | 2.76 [0.30, 5.22] | **0.0283** |
|  | 3 | 1.45 [0.51, 2.39] | **0.0027** | 3.64 [1.18, 6.10] | **0.0038** |
|  | 4 | 1.91 [0.97, 2.85] | **0.0001** | 4.76 [2.30, 7.22] | **0.0002** |
| MnBP | 2 | -0.39 [-1.33, 0.55] | 0.4154 | -1.14 [-3.59, 1.31] | 0.3623 |
|  | 3 | 0.14 [-0.80, 1.08] | 0.7673 | -0.02 [-2.48, 2.44] | 0.9853 |
|  | 4 | 0.67 [-0.28, 1.62] | 0.1657 | 1.41 [-1.08, 3.90] | 0.2684 |
| MEHP | 2 | -0.50 [-1.45, 0.45] | 0.3042 | -0.70 [-3.18, 1.78] | 0.5782 |
|  | 3 | -0.16 [-1.11, 0.79] | 0.7399 | -0.27 [-2.76, 2.22] | 0.8304 |
|  | 4 | -0.07 [-1.01, 0.87] | 0.8814 | -0.14 [-2.61, 2.33] | 0.9146 |
| MEHHP | 2 | 0.79 [-0.16, 1.74] | 0.1062 | 2.22 [-0.27, 4.71] | 0.0810 |
|  | 3 | 1.05 [0.10, 2.00] | **0.0308** | 2.43 [-0.06, 4.92] | 0.0564 |
|  | 4 | 0.92 [-0.05, 1.89] | 0.0644 | 2.42 [-0.11, 4.95] | 0.0612 |
| MEOHP | 2 | 0.57 [-0.37, 1.51] | 0.2369 | 1.21 [-1.26, 3.68] | 0.3374 |
|  | 3 | 1.33 [0.39, 2.27] | **0.0056** | 3.39 [0.94, 5.84] | **0.0071** |
|  | 4 | 0.63 [-0.33, 1.59] | 0.1958 | 1.14 [-1.36, 3.64] | 0.3733 |
| MECPP | 2 | 0.27 [-0.68, 1.22] | 0.5839 | 1.14 [-1.34, 3.62] | 0.3679 |
|  | 3 | 0.99 [0.03, 1.95] | **0.0442** | 3.07 [0.56, 5.58] | **0.0168** |
|  | 4 | 0.87 [-0.08, 1.82] | 0.0725 | 1.79 [-0.69, 4.27] | 0.1575 |
| MBzP | 2 | 0.69 [-0.24, 1.62] | 0.1471 | 1.59 [-0.84, 4.02] | 0.1994 |
|  | 3 | 0.20 [-0.75, 1.15] | 0.6793 | 0.44 [-2.02, 2.90] | 0.7282 |
|  | 4 | 1.74 [0.79, 2.69] | **0.0004** | 4.97 [2.50, 7.44] | **0.0001** |

CI, Confidence Interval. Multivariate associations were tested using Endocrine Disrupting Chemical (EDC) concentrations as quartiles to assess linearity. The model was corrected for age, sex, education, smoking, diabetes status, physical activity and total caloric intake. EDCs which were detected above the limit of quantification (LOQ) in at least 50% of the samples were included in analysis. The lowest quartile (1) was taken as reference group. P-values <0.05 are expressed bold. **Supplementary table 3b.** Multivariate associations between lipid-related traits and urinary paraben, bisphenol and phthalate concentrations expressed as quartiles in the Lifelines population.

|  |  | HDL-cholesterol (mmol/L) | | Triglycerides (mmol/L) | |
| --- | --- | --- | --- | --- | --- |
|  | Quartile | Estimate [CI] | *p*-value | Estimate [CI] | *p*-value |
| MeP | 2 | -0.06 [-0.14, 0.02] | 0.1734 | 0.02 [-0.03, 0.07] | 0.4291 |
|  | 3 | 0.00 [-0.08, 0.08] | 0.9930 | 0.02 [-0.03, 0.07] | 0.4439 |
|  | 4 | -0.03 [-0.11, 0.05] | 0.4257 | 0.05 [0.00, 0.10] | 0.0538 |
| EtP | 2 | -0.01 [-0.09, 0.07] | 0.8250 | 0.03 [-0.02, 0.08] | 0.2797 |
|  | 3 | -0.02 [-0.10, 0.06] | 0.5585 | 0.01 [-0.04, 0.06] | 0.7190 |
|  | 4 | 0.04 [-0.04, 0.12] | 0.2975 | 0.02 [-0.03, 0.07] | 0.3406 |
| PrP | 2 | 0.04 [-0.04, 0.12] | 0.3634 | -0.03 [-0.08, 0.02] | 0.2558 |
|  | 3 | 0.03 [-0.05, 0.11] | 0.4628 | -0.01 [-0.06, 0.04] | 0.6678 |
|  | 4 | -0.02 [-0.10, 0.06] | 0.5948 | 0.03 [-0.02, 0.08] | 0.2439 |
| BPA | 2 | -0.03 [-0.11, 0.05] | 0.4692 | -0.01 [-0.06, 0.04] | 0.7364 |
|  | 3 | -0.04 [-0.12, 0.04] | 0.3029 | 0.00 [-0.05, 0.05] | 0.9062 |
|  | 4 | -0.07 [-0.15, 0.01] | 0.0876 | 0.00 [-0.05, 0.05] | 0.9348 |
| MEP | 2 | -0.07 [-0.15, 0.01] | 0.1040 | 0.00 [-0.05, 0.05] | 0.8969 |
|  | 3 | -0.03 [-0.11, 0.05] | 0.5146 | -0.02 [-0.07, 0.03] | 0.4946 |
|  | 4 | -0.09 [-0.17, -0.01] | **0.0403** | 0.00 [-0.05, 0.05] | 0.9825 |
| MiBP | 2 | 0.00 [-0.08, 0.08] | 0.9714 | 0.03 [-0.02, 0.08] | 0.1819 |
|  | 3 | -0.10 [-0.18, -0.02] | **0.0223** | 0.06 [0.01, 0.11] | **0.0186** |
|  | 4 | -0.04 [-0.12, 0.04] | 0.2965 | 0.02 [-0.03, 0.07] | 0.3533 |
| MnBP | 2 | 0.05 [-0.03, 0.13] | 0.2135 | -0.03 [-0.08, 0.02] | 0.1476 |
|  | 3 | 0.02 [-0.06, 0.10] | 0.6283 | 0.00 [-0.05, 0.05] | 0.9146 |
|  | 4 | 0.01 [-0.07, 0.09] | 0.8213 | -0.03 [-0.08, 0.02] | 0.2508 |
| MEHP | 2 | -0.06 [-0.14, 0.02] | 0.1408 | -0.01 [-0.06, 0.04] | 0.7428 |
|  | 3 | -0.05 [-0.13, 0.03] | 0.2364 | -0.01 [-0.06, 0.04] | 0.6032 |
|  | 4 | -0.01 [-0.09, 0.07] | 0.8863 | -0.07 [-0.12, -0.02] | **0.0053** |
| MEHHP | 2 | -0.03 [-0.11, 0.05] | 0.4135 | 0.06 [0.01, 0.11] | **0.0188** |
|  | 3 | -0.02 [-0.10, 0.06] | 0.6502 | 0.01 [-0.04, 0.06] | 0.6201 |
|  | 4 | 0.00 [-0.08, 0.08] | 0.9551 | -0.01 [-0.06, 0.04] | 0.8259 |
| MEOHP | 2 | -0.05 [-0.13, 0.03] | 0.2060 | 0.04 [-0.01, 0.09] | 0.0682 |
|  | 3 | -0.01 [-0.09, 0.07] | 0.7236 | -0.01 [-0.06, 0.04] | 0.6207 |
|  | 4 | 0.00 [-0.08, 0.08] | 0.9695 | -0.02 [-0.07, 0.03] | 0.4386 |
| MECPP | 2 | 0.02 [-0.06, 0.10] | 0.6046 | -0.01 [-0.06, 0.04] | 0.5994 |
|  | 3 | -0.07 [-0.15, 0.01] | 0.1206 | -0.02 [-0.07, 0.03] | 0.3512 |
|  | 4 | 0.00 [-0.08, 0.08] | 0.9875 | -0.04 [-0.09, 0.01] | 0.0832 |
| MBzP | 2 | -0.03 [-0.11, 0.05] | 0.4625 | 0.01 [-0.04, 0.06] | 0.5680 |
|  | 3 | -0.04 [-0.12, 0.04] | 0.3191 | -0.01 [-0.06, 0.04] | 0.6082 |
|  | 4 | -0.07 [-0.15, 0.01] | 0.1264 | 0.02 [-0.03, 0.07] | 0.5169 |

CI, Confidence Interval. Multivariate associations were tested using Endocrine Disrupting Chemical (EDC) concentrations as quartiles to assess linearity. The model was corrected for age, sex, education, smoking, diabetes status, physical activity and total caloric intake. EDCs which were detected above the limit of quantification (LOQ) in at least 50% of the samples were included in analysis. The lowest quartile (1) was taken as reference group. P-values <0.05 are expressed bold.

|  |  | Glucose (mmol/L) | |
| --- | --- | --- | --- |
|  | Quartile | Estimate [CI] | *p*-value |
| MeP | 2 | 0.07 [-0.03, 0.17] | 0.1585 |
|  | 3 | 0.06 [-0.04, 0.16] | 0.2637 |
|  | 4 | 0.03 [-0.07, 0.13] | 0.5623 |
| EtP | 2 | 0.05 [-0.05, 0.15] | 0.3361 |
|  | 3 | 0.06 [-0.04, 0.16] | 0.2562 |
|  | 4 | -0.03 [-0.13, 0.07] | 0.5885 |
| PrP | 2 | -0.02 [-0.12, 0.08] | 0.6573 |
|  | 3 | 0.01 [-0.09, 0.11] | 0.8474 |
|  | 4 | 0.09 [-0.01, 0.19] | 0.0734 |
| BPA | 2 | -0.04 [-0.14, 0.06] | 0.4518 |
|  | 3 | 0.01 [-0.09, 0.11] | 0.8543 |
|  | 4 | 0.02 [-0.08, 0.12] | 0.7405 |
| MEP | 2 | 0.08 [-0.02, 0.18] | 0.1188 |
|  | 3 | 0.02 [-0.08, 0.12] | 0.7145 |
|  | 4 | 0.03 [-0.07, 0.13] | 0.4868 |
| MiBP | 2 | 0.05 [-0.05, 0.15] | 0.3198 |
|  | 3 | 0.07 [-0.03, 0.17] | 0.1875 |
|  | 4 | 0.00 [-0.10, 0.10] | 0.9300 |
| MnBP | 2 | -0.02 [-0.12, 0.08] | 0.7057 |
|  | 3 | 0.06 [-0.04, 0.16] | 0.2525 |
|  | 4 | -0.03 [-0.13, 0.07] | 0.4834 |
| MEHP | 2 | -0.07 [-0.17, 0.03] | 0.1748 |
|  | 3 | -0.06 [-0.16, 0.04] | 0.2373 |
|  | 4 | -0.08 [-0.18, 0.02] | 0.1088 |
| MEHHP | 2 | 0.02 [-0.08, 0.12] | 0.7422 |
|  | 3 | 0.05 [-0.05, 0.15] | 0.3385 |
|  | 4 | 0.02 [-0.08, 0.12] | 0.7052 |
| MEOHP | 2 | 0.04 [-0.06, 0.14] | 0.3741 |
|  | 3 | 0.06 [-0.04, 0.16] | 0.2286 |
|  | 4 | 0.02 [-0.08, 0.12] | 0.7158 |
| MECPP | 2 | 0.05 [-0.05, 0.15] | 0.3451 |
|  | 3 | 0.03 [-0.07, 0.13] | 0.5142 |
|  | 4 | 0.03 [-0.07, 0.13] | 0.5890 |
| MBzP | 2 | 0.07 [-0.03, 0.17] | 0.1710 |
|  | 3 | -0.02 [-0.12, 0.08] | 0.7082 |
|  | 4 | 0.08 [-0.02, 0.18] | 0.0989 |

**Supplementary table 3c.** Multivariate associations between fasting glucose and urinary paraben, bisphenol and phthalate concentrations expressed as quartiles in the Lifelines population.

CI, Confidence Interval. Multivariate associations were tested using Endocrine Disrupting Chemical (EDC) concentrations as quartiles to assess linearity. The model was corrected for age, sex, education, smoking, diabetes status, physical activity and total caloric intake. EDCs which were detected above the limit of quantification (LOQ) in at least 50% of the samples were included in analysis. All individuals which were diagnosed with diabetes (n = 8), or which had fasting glucose levels ≥ 7.0 mmol/L (n = 41) were excluded from analysis. The lowest quartile (1) was taken as reference group.

**Supplementary table 3d.** Multivariate associations between blood pressure and urinary paraben, bisphenol and phthalate concentrations expressed as quartiles in the Lifelines population.

|  |  | Diastolic blood pressure (mmHg) | | Systolic blood pressure (mmHg) | |
| --- | --- | --- | --- | --- | --- |
|  | Quartile | Estimate [CI] | *p*-value | Estimate [CI] | *p*-value |
| MeP | 2 | -1.04 [-2.94, 0.86] | 0.2835 | -0.71 [-3.37, 1.95] | 0.6015 |
|  | 3 | -0.07 [-1.97, 1.83] | 0.9415 | -1.04 [-3.71, 1.63] | 0.4467 |
|  | 4 | -0.23 [-2.18, 1.72] | 0.8190 | -1.09 [-3.81, 1.63] | 0.4341 |
| EtP | 2 | 0.65 [-1.26, 2.56] | 0.5013 | 3.67 [1.03, 6.31] | **0.0068** |
|  | 3 | 0.40 [-1.51, 2.31] | 0.6817 | 0.30 [-2.35, 2.95] | 0.8248 |
|  | 4 | -0.37 [-2.3, 1.56] | 0.7058 | -0.66 [-3.33, 2.01] | 0.6263 |
| PrP | 2 | -0.41 [-2.31, 1.49] | 0.6688 | 0.32 [-2.34, 2.98] | 0.8149 |
|  | 3 | 1.24 [-0.67, 3.15] | 0.2059 | 0.76 [-1.92, 3.44] | 0.5795 |
|  | 4 | 0.34 [-1.57, 2.25] | 0.7256 | 0.13 [-2.55, 2.81] | 0.9235 |
| BPA | 2 | 0.45 [-1.47, 2.37] | 0.6452 | 1.68 [-1.01, 4.37] | 0.2197 |
|  | 3 | 0.45 [-1.45, 2.35] | 0.6448 | 1.58 [-1.08, 4.24] | 0.2442 |
|  | 4 | -0.34 [-2.23, 1.55] | 0.7222 | 0.73 [-1.92, 3.38] | 0.5893 |
| MEP | 2 | -0.23 [-2.13, 1.67] | 0.8126 | -0.39 [-3.04, 2.26] | 0.7762 |
|  | 3 | -0.09 [-2.02, 1.84] | 0.9310 | 0.72 [-1.98, 3.42] | 0.6001 |
|  | 4 | -0.27 [-2.19, 1.65] | 0.7786 | 0.16 [-2.52, 2.84] | 0.9092 |
| MiBP | 2 | 0.35 [-1.57, 2.27] | 0.7195 | 1.68 [-0.99, 4.35] | 0.2197 |
|  | 3 | 1.21 [-0.71, 3.13] | 0.2184 | 3.48 [0.81, 6.15] | **0.0110** |
|  | 4 | 0.00 [-1.92, 1.92] | 0.9990 | 2.72 [0.04, 5.40] | **0.0465** |
| MnBP | 2 | -2.37 [-4.25, -0.49] | **0.0138** | -1.82 [-4.47, 0.83] | 0.1789 |
|  | 3 | -1.62 [-3.51, 0.27] | 0.0930 | -1.14 [-3.79, 1.51] | 0.3983 |
|  | 4 | 0.25 [-1.66, 2.16] | 0.7965 | 0.96 [-1.73, 3.65] | 0.4840 |
| MEHP | 2 | -0.39 [-2.3, 1.52] | 0.6899 | 0.40 [-2.27, 3.07] | 0.7713 |
|  | 3 | -0.81 [-2.73, 1.11] | 0.4102 | 0.13 [-2.56, 2.82] | 0.9269 |
|  | 4 | -0.37 [-2.27, 1.53] | 0.7027 | -0.44 [-3.11, 2.23] | 0.7478 |
| MEHHP | 2 | -0.98 [-2.91, 0.95] | 0.3211 | -0.19 [-2.89, 2.51] | 0.8922 |
|  | 3 | -0.59 [-2.52, 1.34] | 0.5461 | -0.35 [-3.05, 2.35] | 0.7981 |
|  | 4 | -0.56 [-2.52, 1.40] | 0.5773 | -0.54 [-3.28, 2.20] | 0.7013 |
| MEOHP | 2 | -0.22 [-2.13, 1.69] | 0.8221 | 0.86 [-1.82, 3.54] | 0.5308 |
|  | 3 | -0.19 [-2.09, 1.71] | 0.8475 | -0.08 [-2.75, 2.59] | 0.9545 |
|  | 4 | -0.99 [-2.93, 0.95] | 0.3195 | -0.30 [-3.01, 2.41] | 0.8264 |
| MECPP | 2 | 0.43 [-1.49, 2.35] | 0.6611 | 0.44 [-2.25, 3.13] | 0.7467 |
|  | 3 | -0.97 [-2.91, 0.97] | 0.3295 | -1.40 [-4.12, 1.32] | 0.3150 |
|  | 4 | -0.91 [-2.83, 1.01] | 0.3561 | -1.05 [-3.74, 1.64] | 0.4438 |
| MBzP | 2 | 1.09 [-0.81, 2.99] | 0.2606 | 2.59 [-0.06, 5.24] | 0.0560 |
|  | 3 | -0.47 [-2.4, 1.46] | 0.6322 | 0.37 [-2.32, 3.06] | 0.7889 |
|  | 4 | -0.39 [-2.32, 1.54] | 0.6965 | 2.37 [-0.33, 5.07] | 0.0860 |

CI, Confidence Interval. Multivariate associations were tested using Endocrine Disrupting Chemical (EDC) concentrations as quartiles to assess linearity. The model was corrected for age, sex, education, smoking, diabetes status, physical activity and total caloric intake. EDCs which were detected above the limit of quantification (LOQ) in at least 50% of the samples were included in analysis. The lowest quartile (1) was taken as reference group. P-values <0.05 are expressed bold.
